# Continuous Casting Slab Mold: Key Role of Nozzle Immersion Depth

**DOI:** 10.3390/ma17194888

**Published:** 2024-10-05

**Authors:** Liang Chen, Xiqing Chen, Pu Wang, Jiaquan Zhang

**Affiliations:** School of Metallurgical and Ecological Engineering, University of Science and Technology Beijing, No. 30 Xueyuan Road, Haidian District, Beijing 100083, China

**Keywords:** slab mold, nozzle immersion depth, flow field, slag entrapment, initial solidification

## Abstract

Based on a physical water model with a scaling factor of 0.5 and a coupled flow–heat transfer–solidification numerical model, this study investigates the influence of the submerged entry nozzle (SEN) depth on the mold surface behavior, slag entrapment, internal flow field, temperature distribution, and initial solidification behavior in slab casting. The results indicate that when the SEN depth is too shallow (80 mm), the slag layer on the narrow face is thin, leading to slag entrapment. Within a certain range of SEN depths (less than 170 mm), increasing the SEN depth reduces the impact on the mold walls, shortening the “plateau period” of stagnated growth on the narrow face shell. This allows the upper recirculation flow to develop more fully, resulting in an increase in the surface flow velocity and an expansion in the high-temperature region near the meniscus, which promotes uniform slag melting but also heightens the risk of slag entrainment due to shear stress at the liquid surface (with 110 mm being the most stable condition). As the SEN depth continues to increase, the surface flow velocity gradually decreases, and the maximum fluctuation in the liquid surface diminishes, while the full development of the upper recirculation zone leads to a higher and more uniform meniscus temperature. This suggests that in practical production, it is advisable to avoid this critical SEN depth. Instead, the immersion depth should be controlled at a slightly shallower position (around 110 mm) or a deeper position (around 190 mm).

## 1. Introduction

In recent years, continuous casting has become a widely adopted method in modern steel production across the globe [1]. As the demand for higher cleanliness in sheet products increases, greater emphasis has been placed on improving continuous casting processes to produce cleaner slab materials [2]. Numerous studies have demonstrated [3,4,5,6,7] that the surface quality of slabs is closely linked to the flow behavior within the mold. To optimize the metallurgical performance of slab casting molds, many researchers have made significant contributions by investigating factors such as the inner diameter of the submerged entry nozzle (SEN) [8], the shape of the side ports [9,10,11,12], the inclination angle of the side ports [13,14,15,16], the shape of the nozzle bottom [17], the casting speed [18], and the argon gas flow rate [19]. These studies have led to a substantial consensus in the field, which will not be reiterated here.

The submerged entry nozzle (SEN) depth refers to the distance from the mold surface to the center of the SEN side ports. While the influence of the SEN depth on mold metallurgical performance has been widely discussed, it remains a topic of some controversy. Hoffken et al. [20] suggested that increasing the SEN depth reduces the strength of the upper recirculation flow, thereby decreasing liquid surface fluctuations and stabilizing the steel–slag interface. They identified an optimal range of SEN depths that minimizes the likelihood of longitudinal cracks in the cast slab. Miranda et al. [21], through physical and numerical simulations, demonstrated that as the SEN depth increases, the upper recirculation flow develops more fully, resulting in a higher surface flow velocity. Zhang [22] also found that with an increasing SEN depth, the surface flow velocity of molten steel in the mold initially increases and then decreases. Wang et al. [23] observed that as the SEN depth increases, the number of argon bubbles near the steel–slag interface rises, exacerbating liquid level fluctuations that should otherwise be reduced. Using a water model combined with tracer injection, particle image velocimetry, and ultrasonic Doppler techniques, Ramos [24] studied the jet flow patterns inside the mold at two different SEN depths. Saldaña-Salas [25] investigated the effects of the SEN depth on hydrodynamic structure, free surface oscillation, and slag layer opening, finding that working at SEN depths of 100 mm or less may be detrimental to steel quality as it promotes Stokes surface oscillation and excessive slag layer opening. Xu et al. [26] found that the smaller the SEN depth, the more severe the slag entrapment, and the less stable the liquid surface becomes when the casting speed fluctuates. The primary point of contention centers around the impact of the SEN depth on the surface flow velocity within the mold.

Conventionally, it is understood that as the submerged entry nozzle (SEN) depth increases, the influence of the upper recirculation flow on the meniscus weakens, leading to reduced mold surface flow velocity and fluctuations, thereby lowering the risk of slag entrapment. However, this study investigates flow patterns and slag entrapment behavior using a water model for a slab mold with a section size of 220 mm × 1600 mm in an online adjustable-width mold (220 mm × 1600~2300 mm). Additionally, this study examines the variations in the flow field, temperature distribution, and initial solidification within a mold with a conventional section size of 220 mm × 2065 mm. The findings reveal that within a certain range of SEN depths, increasing the SEN depth can actually increase the surface flow velocity, thereby heightening the risk of slag entrapment. Therefore, a thorough understanding of the critical role of the SEN depth in continuous casting molds is of great importance. This research aims to provide valuable reference data and theoretical guidance for recognizing and selecting the optimal SEN depth in the actual continuous casting process of slabs.

## 2. Establishment of Physical Model and Mathematical Model

### 2.1. Establishment of Physical Model

Due to limitations of the on-site experimental apparatus, the smallest cross-section was selected for the physical simulation to analyze the flow patterns and slag entrapment behavior. The physical model corresponds to a slab mold with a cross-section of 1600 mm × 220 mm and an effective length of 900 mm, and a scale factor of λ = 0.5 was applied. To prevent backflow at the model’s outlet, the height of the model was extended to 900 mm, and a perforated plate along with a calm water tank was installed at the mold exit. The hydraulic experiments were conducted based on similarity principles [27,28,29]. The fundamental requirement for achieving similarity between the model and the prototype is both geometric and dynamic similarity. The flow conditions to be reproduced include turbulence, flow patterns, and fluctuations. The forces responsible for these phenomena are inertia, viscosity, gravity, and surface tension. To achieve dynamic similarity between the model and the prototype, the ratio of these forces in both systems must be identical. Since most of the molten steel is in a turbulent state, with Reynolds numbers far exceeding the second critical value, it is sufficient to consider cases where the Froude and Weber numbers are equal [30].

Considering the similarity condition of the model flow in which the Froude numbers are equal, the following can be obtained:

(1) $$Fr_{p}=Fr_{m}$$

(2) $$\;{\left(u^{2}/gL\right)}_{m}={\left(u^{2}/gL\right)}_{P}$$

(3) $$\;u_{m}/u_{p}=L_{m}^{\frac{1}{2}}/L_{m}^{\frac{1}{2}}=\lambda^{\frac{1}{2}}$$

(4) $$\;Q_{m}/Q_{p}=u_{m}L_{m}^{2}/u_{p}L_{p}^{2}=\lambda^{\frac{5}{2}}$$

In the equations, the subscript m denotes the model, while p represents the prototype. *L* stands for the characteristic length (m), *u* is the flow velocity (m·s^−1^), *g* is the gravitational acceleration (m·s^−2^), and *Q* is the flow rate (m^3^/h).

Considering the similarity condition of the slag–metal interface in which the Weber numbers are equal, the following can be obtained:

(5) $$We_{p}=We_{m}$$

(6) $${\left(\frac{\rho u^{2}L}{\sigma }\right)}_{p}={\left(\frac{\rho u^{2}L}{\sigma }\right)}_{m}$$

A dimensional analysis reveals that, in order to simulate the flow behavior of slag and molten steel, the dynamic viscosity (*μ*) and density (*ρ*) of the medium used to represent the protective slag must satisfy the following conditions:

(7) $$\mu_{slag}/\mu_{steel}=\mu_{oil}/\mu_{water}$$

(8) $$\rho_{slag}/\rho_{steel}=\rho_{oil}/\rho_{water}$$

The relationship satisfied by the kinematic viscosity *ν* of the material used to simulate the mold flux can be obtained [2,26,29]:

(9) $$\;v_{slag}/v_{steel}=v_{oil}/v_{water}$$

In the equation, *ρ* represents the fluid density (kg·m^−3^), *σ* is the surface tension coefficient (N·m^−1^), *μ* is the dynamic viscosity (kg·m^−1^·s^−1^), and *ν* denotes the kinematic viscosity (m^2^·s^−1^).

In this study, a mixture of kerosene and 68# vacuum pump oil was used to simulate mold slag. By adjusting the ratio of the two oils, the optimal mixture with the most suitable kinematic viscosity was identified to simulate the distribution of mold slag at the slag–steel interface [26,29]. During the experiments, the slag layer thickness was consistently maintained at 15 mm for each test.

**Figure 1:**
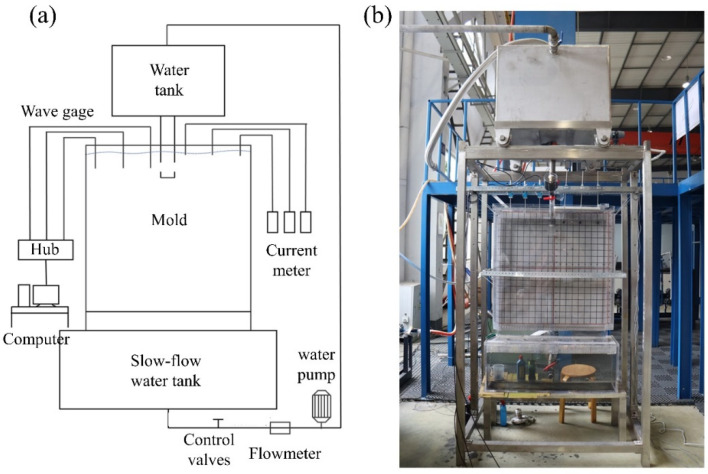
Physical model experimental device: (**a**) schematic diagram; (**b**) physical map.

**Figure 2:**
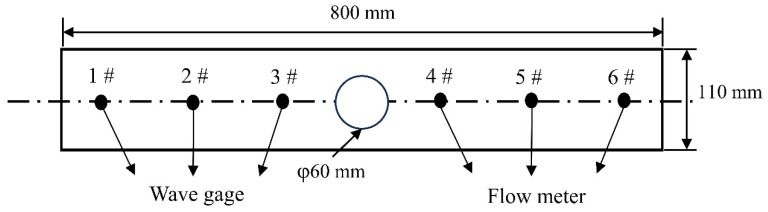
Position diagram of liquid level fluctuation (1#–3#) and surface velocity measurement (4#–6#).

Figure 1 shows the water model experimental device, including the main body of the mold model, the water circulation system, the data acquisition system, and the image recording system. During the experiments, to ensure stable flow from the submerged entry nozzle (SEN), the tundish level was consistently maintained at the overflow level. The water flow rate was adjusted using an electromagnetic valve at the SEN, while a water pump was used to extract water according to the inflow rate. Once the inflow and outflow rates were balanced and the mold model’s liquid level was stabilized, the liquid surface wave height was monitored using a CBG03 capacitive digital wave height meter, with data being recorded for 180 s. The data were processed to obtain a significant wave height, defined as one-third of the highest wave values, with three data segments being analyzed per experiment. The average of these segments was taken as the final value. The surface flow velocity was measured using an LGY-2 flowmeter, with the velocity at each measurement point being recorded continuously for 60 s. The measurements were repeated three times, and the average value was calculated. To observe the wave height and surface flow velocity near the SEN, the mold walls, and at the 1/4 width position of the mold, three measurement points were selected on each side of the mold, as illustrated in Figure 2.

### 2.2. Establishment of Mathematical Model

#### 2.2.1. Assumption

In the numerical simulation process, to realistically replicate the complex and interrelated metallurgical phenomena occurring during continuous casting while also improving computational efficiency, the following basic assumptions were made:

1. The flow of molten steel is regarded as a steady state, and the molten steel is treated as an incompressible Newtonian fluid, with constant viscosity, specific heat capacity, and thermal conductivity.
2. The turbulence model employs the low-Reynolds-number *k-ε* model.
3. The flow field within the slab mold is assumed to be symmetric with respect to the central plane along the wide face.
4. The effects of molten steel solidification shrinkage, mold powder, and mold oscillation on the flow and temperature fields of the molten steel are neglected.
5. The mushy zone is treated as a porous medium, and the internal flow follows Darcy’s law.

#### 2.2.2. Governing Equation

Based on the aforementioned basic assumptions, a coupled model of flow, heat transfer, and solidification within the slab mold was established. The governing equations for the continuous phase include the continuity equation and the momentum equation. Turbulent flow is modeled using the low-Reynolds-number turbulence model. The governing equations for the model are as follows [31,32,33,34]:

(1) Continuity equation:

(10) $$\frac{\partial \left(\rho u_{j}\right)}{\partial x_{j}}=0$$

where *ρ* is the density of molten steel, kg·m^−3^; *u_j_* is the velocity of the fluid in the *j* direction, m/s.

(2) Momentum equation:

(11) $$\frac{\partial \left(\rho u_{i}\right)}{\partial t}+\rho \frac{\partial \left(u_{i}u_{j}\right)}{\partial x_{j}}=-\frac{\partial p}{\partial x_{i}}+\frac{\partial }{\partial x_{j}}\left[\mu_{\mathrm{eff}}\left(\frac{\partial u_{i}}{\partial x_{j}}+\frac{\partial u_{j}}{\partial x_{i}}\right)\right]+\rho \vec{g}+F_{B}+S_{P}$$

where *u_j_* is the velocity of fluid in the *j* direction, m/s; *p* is pressure, Pa; and *S_p_* is the source term of porous media motion. The turbulence characteristics of the interdendritic fluid are enhanced by adding the source term of porous media. *μ*_eff_ is the effective viscosity coefficient:

(12) $$S_{p}=\frac{{\left(1-f_{L}\right)}^{2}}{{f_{L}}^{3}+\zeta }A_{mush}(v-v_{c})$$

(13) $$\mu_{\mathrm{eff}}=\mu_{l}+\mu_{t}=\mu_{l}+\rho c_{\mu }\frac{\varepsilon^{2}}{k}$$

where *A_mush_* is the constant for the mushy zone, set to 1 × 10^8^. To prevent the denominator from reaching zero, *ζ* is assigned a small value (0.001); *v* represents the molten steel flow velocity, m/s; *v*_c_ is the casting speed, m/s; *f_L_* denotes the liquid fraction; *μ_l_* is laminar viscosity, kg·m^−1^·s^−1^; *μ_t_* is turbulent viscosity, kg·m^−1^·s^−1^; *k* is turbulent kinetic energy, m^2^·s^−2^; and *ε* is the turbulent kinetic energy dissipation rate, m^2^·s^−3^.

F_B_ is thermal buoyancy, which is usually calculated using the Boussinesq hypothesis [34]:

(14) $$F_{B}=\rho g_{i}\beta (T-T_{l})$$

where *β* is the coefficient of thermal expansion, K^−1^; T is the local temperature, K; and T_l_ is the liquidus temperature, K.

(3) Low Reynolds number *k-ε* equation

Since the solid–liquid interface during solidification cannot be predicted in advance, the low-Reynolds-number *k-ε* turbulence model is better suited for calculating the flow solidification behavior in continuous casting compared to the standard *k-ε* turbulence model [31].

Turbulent kinetic energy equation:

(15) $$\rho \frac{\partial \left(ku_{i}\right)}{\partial x_{i}}=\frac{\partial }{\partial x_{i}}\left[\left(\mu_{l}+\frac{\mu_{t}}{\sigma_{k}}\right)\frac{\partial k}{\partial x_{i}}\right]+G_{k}-\rho \varepsilon +\rho D+S_{k}$$

Turbulent kinetic energy dissipation rate equation:

(16) $$\rho \frac{\partial \left(\varepsilon u_{i}\right)}{\partial x_{i}}=\frac{\partial }{\partial x_{i}}\left[\left(\mu_{l}+\frac{\mu_{t}}{\sigma_{\varepsilon }}\right)\frac{\partial \varepsilon }{\partial x_{i}}\right]+C_{1\varepsilon }\frac{\varepsilon }{k}\cdot G_{k}-C_{2\varepsilon }\rho \frac{\varepsilon^{2}}{k}+\rho E+S_{\varepsilon }$$

The turbulent viscosity *μ*_t_ is calculated using the Kolmogorov–Prandtl relation.

(17) $$\mu_{t}=\rho f_{\mu }C_{\mu }\frac{k^{2}}{\varepsilon }$$

(18) fμ=exp−2.5/1+Ret502

(19) Ret=ρk2μlε

(20) $$D=2\mu_{l}{\left(\frac{\partial \left(\sqrt{k}\right)}{\partial x_{j}}\right)}^{2}$$

(21) $$E=2\frac{\mu_{l}\mu_{t}}{\rho }{\left(\frac{\partial^{2}u_{i}}{\partial x_{j}\partial x_{k}}\right)}^{2}$$

(22) $$G_{k}=\mu_{t}\frac{\partial u_{j}}{\partial x_{i}}\left(\frac{\partial u_{i}}{\partial u_{j}}+\frac{\partial u_{j}}{\partial u_{i}}\right)$$

*S_k_* and *S_ε_* are the source terms of the *k* equation and the *ε* equation caused by the solidification of molten steel, respectively.

(23) $$S_{k}=\frac{{\left(1-f_{L}\right)}^{2}}{\left({f_{L}}^{3}+\xi \right)}A_{mush}k$$

(24) $$S_{\varepsilon }=\frac{{\left(1-f_{L}\right)}^{2}}{\left({f_{L}}^{3}+\xi \right)}A_{mush}\varepsilon$$

where *G*_k_ is the turbulent kinetic energy generation term; *C*_1ε_, *C*_2ε_, *C_μ_*, *σ*_k_, and *σ*_ε_ are empirical constants, and the values recommended by Launder and Spalding [32] are 1.44, 1.92, 0.09, 1.0, and 1.3, respectively. *A_mush_* is the coefficient of the mushy zone.

(4) Energy equation:

(25) $$\rho u_{i}\frac{\partial H}{\partial x_{i}}=\frac{\partial }{\partial x_{i}}\left[\left(k_{l}+\frac{\mu_{t}}{pr_{t}}\right)\frac{\partial T}{\partial x_{i}}\right]$$

where *k_t_* is the thermal conductivity of molten steel laminar flow, W·m^−1^·k^−1^; *Pr_t_* is the turbulent Prandtl number, which is set to 0.85 in the model; and *H* is the total enthalpy of the system, which is composed of apparent enthalpy and latent enthalpy.

#### 2.2.3. Mesh and Boundary Conditions

**Figure 3:**
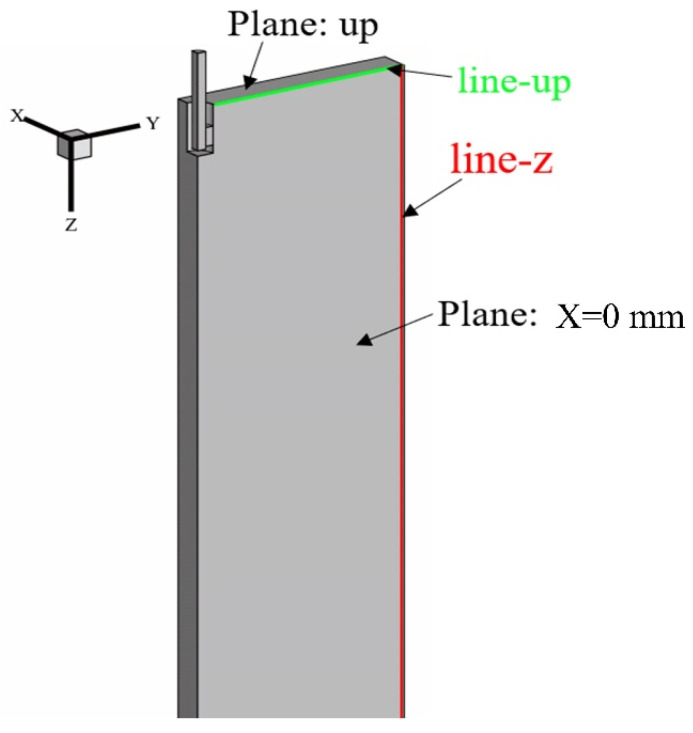
Geometric model of mold calculation domain.

Since water modeling cannot account for temperature and initial solidification behavior, the numerical simulation focuses on a slab continuous casting mold with a typical section size of 2065 mm × 220 mm from a steel plant. Considering the geometric symmetry of the mold, a quarter-geometry model was used for the simulation. Figure 3 shows the geometric model of the mold’s computational domain along with the characteristic lines and cross sections used for the numerical analysis. In this coordinate system, the *Z*-axis corresponds to the casting direction, the *Y*-axis to the wide face direction, and the *X*-axis to the narrow face direction of the mold.

**Figure 4:**
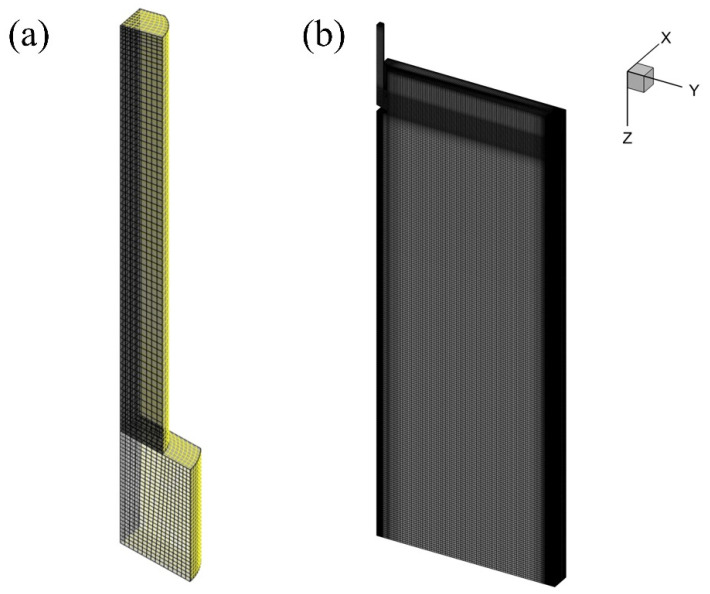
Submerged entry nozzle and mold grid division diagram: (**a**) submerged entry nozzle; (**b**) mold calculation domain.

To ensure the continuity of molten steel flow within the mold and as it exits during the actual continuous casting solidification process, an effective computational length of 2710 mm was selected, encompassing the mold, foot roll section, and part of the bending section. The model was meshed using hexahedral elements in Ansys Fluent’s ICEM, with appropriate block structuring being applied to the physical model during the meshing process. The mesh parameters were carefully controlled during the segmentation process to achieve a higher quality and more realistic results. The mesh around the mold walls and the solidifying shell was refined. The final mesh for the mold and SEN is shown in Figure 4, with a total mesh count of approximately 1.7 million.

Considering the insulating effect of the mold powder, the free surface was treated as an adiabatic wall with zero shear stress [34,35]. A velocity inlet boundary condition was applied, with the inlet temperature set to the casting temperature. The inlet velocity was determined based on the mass conservation principle of continuous casting converted from the casting speed.

(26) $$v_{0}=\frac{v_{c}\times S_{out}}{S_{in}}$$

The calculation formulas of the turbulent kinetic energy *k* and turbulent dissipation rate *ε* are as follows:

(27) $$k=0.01{v_{0}}^{2}$$

(28) $$\varepsilon =\frac{2k^{1.5}}{D_{1}}$$

where *v*_0_ is the inlet velocity, m/s; *v_c_* is the casting speed, m/s; *S_out_* is the cross-sectional area of the slab, m^2^; *S_in_* is the cross-sectional area of the submerged nozzle, m^2^; and *D*_1_ is the hydraulic diameter at the entrance, m.

At the outlet of the computational domain, a fully developed boundary condition was applied, meaning that the gradients of all physical quantities (pressure and velocity) along the outlet normal direction were set to zero. On the symmetry planes, the normal gradients of velocity, temperature, and all other physical quantities were also set to zero.

All walls were modeled as no-slip boundaries, with the SEN walls being treated as adiabatic. The mold walls and the secondary cooling zone were assigned specified values for heat flux, convective heat transfer coefficient, and radiative heat transfer coefficient. The heat flux distribution on the wide and narrow faces of the mold was based on the equations proposed by Savage and Pritchard [36]:

(29) $$\bar{q}=\frac{c_{w}\cdot m\cdot \Delta T}{S_{eff}}$$

(30) $$b=\frac{1.5\times (2,680,000-\bar{q})}{\sqrt{L_{m}/v_{c}}}$$

(31) $$q=2,680,000-b\sqrt{L/v_{c}}$$

Secondary cooling zone [37]:

(32) $$h_{1}=1570\times W^{0.55}(1-0.0075T_{0})/\alpha$$

where $\bar{q}$ is the average heat flux, W m^−2^; *L* is the distance from the meniscus, m; *L*_m_ is the effective length of mold, m; *q* is the heat flux of strand surface, W m^−2^; *C*_w_ is the specific heat capacity of cooling water, J kg^−1^ K^−1^; *m* is the cooling water flow rate of mold, kg s^−1^; Δ*T* is the temperature difference in cooling water, K; *S*_eff_ is the effective cooling area, m^2^; *h*_1_ is the comprehensive heat transfer coefficient of the surface, W m^−2^ K^−1^; *W* is the cooling water volume flow rate, L m^−2^ s^−1^; *T*_0_ is the ambient temperature, K; and α is the correction factor.

For detailed validation of this model, refer to our team’s recently published research [38]. The model is reliable, accurate, and suitable for the analysis presented in this paper.

### 2.3. Simulation Conditions Parameters

All parameters used in the numerical calculations were either directly obtained from the actual process parameters or calculated based on them. The specific structural and process parameters involved in the numerical simulation are listed in Table 1.

Additionally, the material properties used in the numerical simulation are listed in Table 2. The solid phase density of the steel is 7400 kg/m^3^, and the liquid phase density is 7020 kg/m^3^. The density in the mushy zone is taken as the average density, 7200 kg/m^3^. The liquidus and solidus temperatures of the steel are determined by its chemical composition. The liquidus and solidus temperatures were calculated using empirical formulas based on the target contents of elements such as C, Si, Mn, P, S, and Cu in the steel. The calculated results are generally consistent with those obtained using thermodynamic software.

## 3. Result and Discussion

### 3.1. Analysis of Water Modeling Results for 1600 mm × 220 mm Section

**Figure 5:**
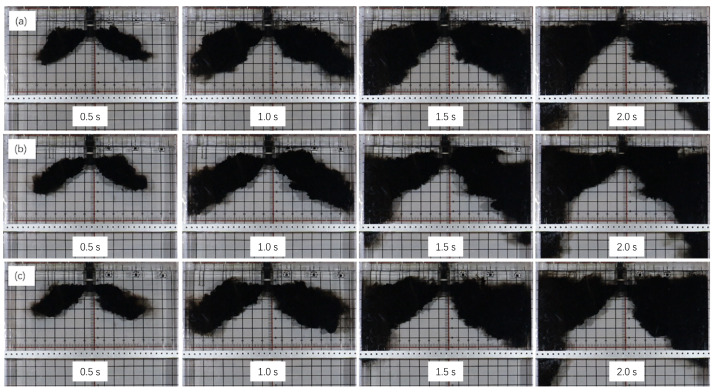
Different SEN depths of molten steel flow trajectory tracer: (**a**) 80 mm; (**b**) 110 mm; and (**c**) 140 mm.

Figure 5 shows the flow trajectories of molten steel at different times, which were obtained using a tracer in the water model at various submerged entry nozzle depths. It is evident that as the SEN depth increases, the upper recirculation zone gradually expands, and the jet impingement point moves progressively downward.

**Figure 6:**
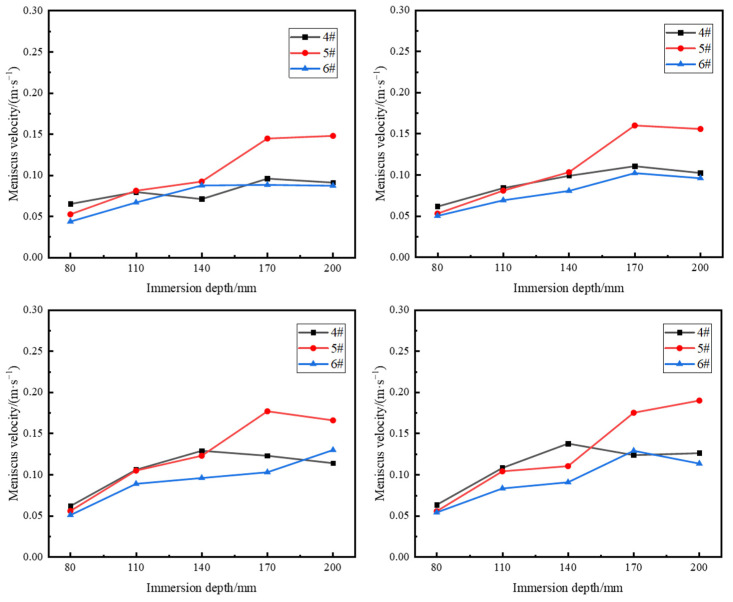
Surface velocity distribution curves at different SEN depths.

Figure 6 presents the results of the water model experiments, showing the surface flow velocity distribution at different SEN depths for casting speeds of 0.9 m/min, 1.0 m/min, 1.1 m/min, and 1.2 m/min. It can be observed that as the SEN depth increased from 80 mm to 170 mm, the surface flow velocity at the 1/4 position of the mold continued to increase. However, when the SEN depth further increased to 200 mm, the rate of increase in flow velocity diminished or even gradually decreased. Near the SEN and the narrow face, the flow velocity first increased and then decreased. This is because as the SEN depth increases within a certain range, the upper recirculation zone expands, providing sufficient space for the upper recirculation to fully develop before entering the horizontal dissipation zone, thereby increasing the surface flow velocity. However, with further increases in the SEN depth, the distance that the upper recirculation must travel to reach the meniscus becomes longer, resulting in greater kinetic energy dissipation and a subsequent gradual decrease in the surface flow velocity.

**Figure 7:**
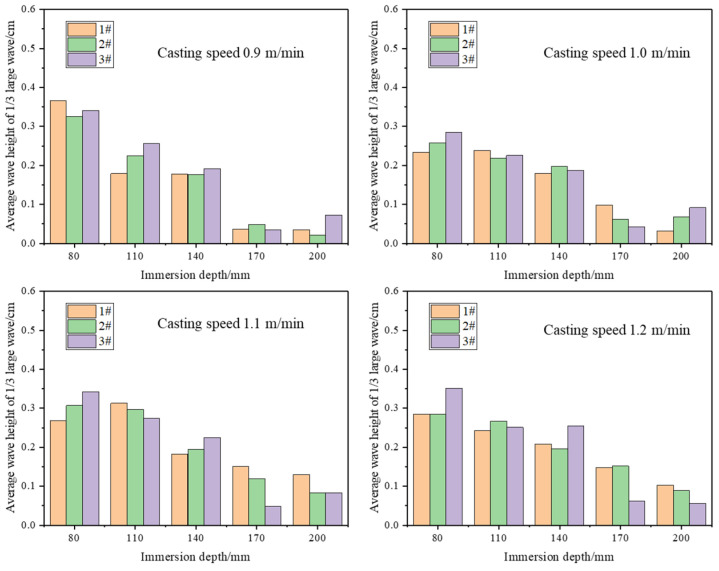
Effects of different SEN depths on liquid level fluctuation.

Figure 7 shows the results of the water model experiments on liquid surface fluctuations at different SEN depths for casting speeds of 0.9 m/min, 1.0 m/min, 1.1 m/min, and 1.2 m/min. As the SEN depth increased from 80 mm to 200 mm, liquid surface fluctuations gradually decreased. When the SEN depth is shallow, the impingement depth is small, and with the same outlet flow velocity, the upper recirculation loses less kinetic energy as it reaches the meniscus. This results in a higher vertical (z-direction) velocity on the mold surface, leading to greater surface disturbances. This is manifested as severe surface fluctuations, which increases the risk of slag entrapment in the mold.

**Figure 8:**
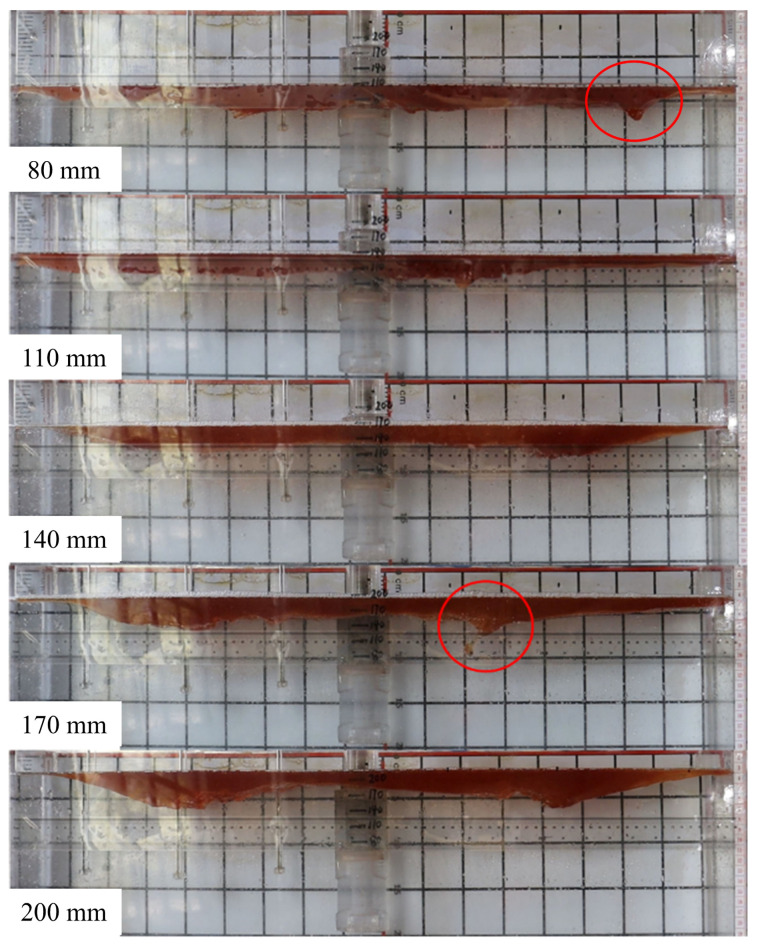
Mold flux coverage at different SEN depths at casting speed of 1.1 m/min.

Figure 8 illustrates the coverage of mold flux on the mold surface at different SEN depths. It can be observed that the mold flux layer on the narrow face is the thinnest, posing a high risk of surface exposure, while the flux layer at the 1/4 position on the wide face is the thickest, making this area prone to shear-induced slag entrapment. When the SEN depth is 80 mm, shear-induced slag entrapment occurs on the narrow face due to excessive liquid surface fluctuations. As the SEN depth increases, the mold flux at the 1/4 position on the wide face gradually thickens. At an SEN depth of 170 mm, significant shear-induced slag entrapment is observed at the 1/4 position on the wide face, attributed to the excessive flow velocity. In comparison, at an SEN depth of 110 mm, the flux layer thickness is most evenly distributed.

### 3.2. Analysis of Numerical Simulation Results for 2065 mm × 220 mm Section

**Figure 9:**
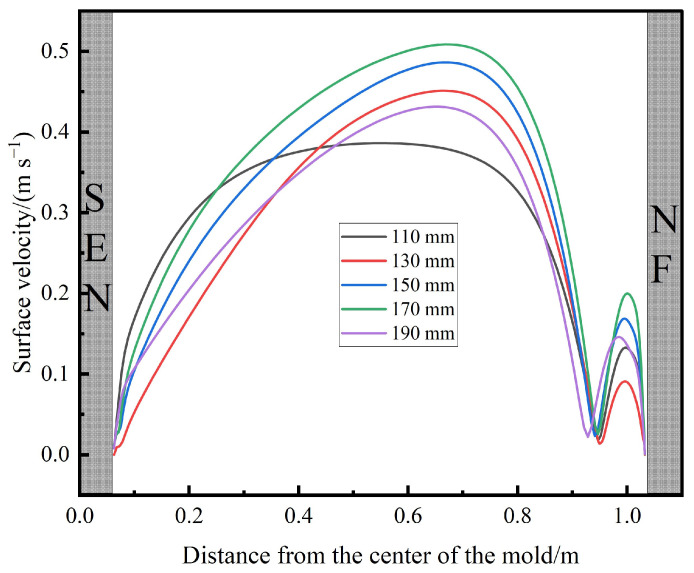
Velocity distribution on line-up at different SEN depths.

Figure 9 illustrates the impact of the SEN depth on the velocity distribution of the free surface in the mold. As shown in the figure, when the SEN depth increases from 110 mm to 170 mm, the maximum surface velocity rises from 0.386 m/s to 0.509 m/s. However, as the SEN depth further increases to 190 mm, the maximum surface velocity decreases to 0.431 m/s. The maximum surface velocity consistently occurs at the 1/4 position of the mold. This indicates the presence of a critical SEN depth at which the surface velocity reaches its peak.

**Figure 10:**
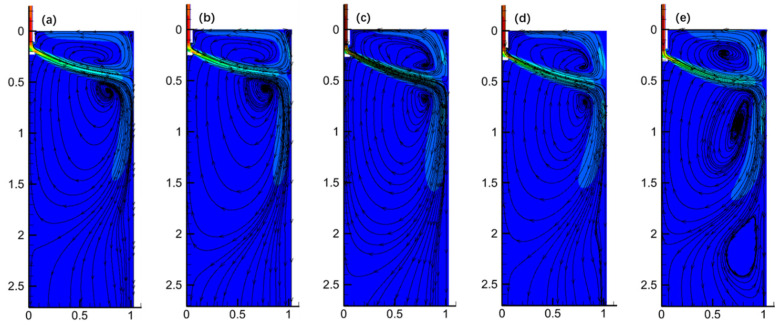
Velocity distribution and streamline diagram of characteristic surface X = 0 m: (**a**) SEN depth of 110 mm; (**b**) SEN depth of 130 mm; (**c**) SEN depth of 150 mm; (**d**) SEN depth of 170 mm; and (**e**) SEN depth of 190 mm.

Figure 10 shows the velocity distribution and streamlines of molten steel at the X = 0 m plane in the mold at different SEN depths. It can be observed that the flow pattern of molten steel in the mold remains consistent across different SEN depths, comprising three main regions: the jet zone, the upper recirculation zone, and the lower recirculation zone. However, as the SEN depth increases, the jet impingement point on the narrow face gradually moves downward. Additionally, it is observed that the vortex in the upper recirculation zone evolves from a less distinct circulation to a well-defined vortex with a centered vortex core, indicating the progressive development and completion of the upper recirculation flow [21,22].

**Figure 11:**
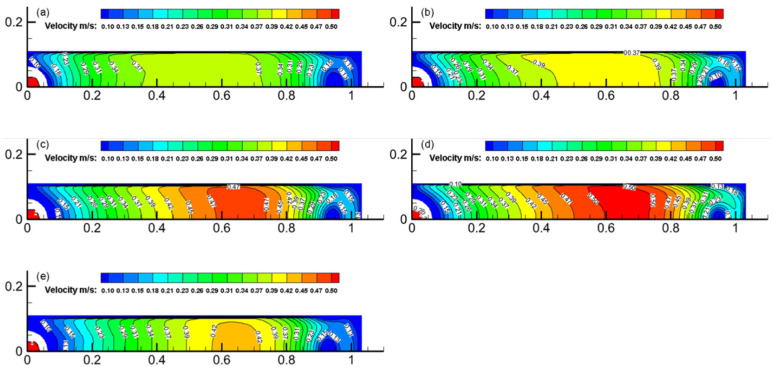
Velocity contour of free surface: (**a**) SEN depth of 110 mm; (**b**) SEN depth of 130 mm; (**c**) SEN depth of 150 mm; (**d**) SEN depth of 170 mm; and (**e**) SEN depth of 190 mm.

Figure 11 presents the velocity contour plots of the free surface in the mold at different SEN depths. It can be seen that at an SEN depth of 110 mm, there is no distinct region with a maximum velocity (>0.4 m/s). This is because, at a shallower SEN depth, the upper recirculation zone is relatively narrow, and due to the tangential forces of the liquid surface, the upward flow stream enters the horizontal dissipation zone before the recirculation can fully develop, resulting in lower surface velocities in this region. However, as the SEN depth increases to 170 mm, the upper recirculation zone becomes more fully developed, leading to an increase in the maximum surface velocity, with the location of this maximum velocity gradually shifting towards the narrow face of the mold. When the SEN depth is further increased to 190 mm, despite the more complete development of the upper recirculation zone, the molten steel’s momentum has to travel a longer distance to reach the surface, leading to greater momentum loss, which, in turn, causes the maximum surface velocity to be lower than that at the 170 mm SEN depth.

**Figure 12:**
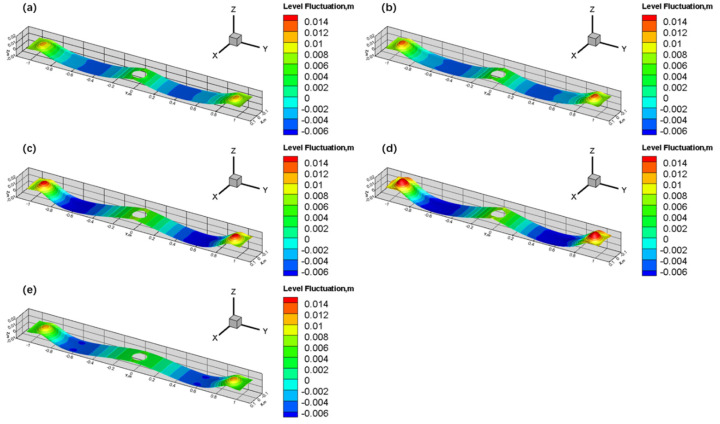
Fluctuations in mold free surface: (**a**) SEN depth of 110 mm; (**b**) SEN depth of 130 mm; (**c**) SEN depth of 150 mm; (**d**) SEN depth of 170 mm; and (**e**) SEN depth of 190 mm.

Figure 12 illustrates the fluctuations in the free surface in the mold at different SEN depths. The fluctuations in the molten steel at the free surface can be described by the relative height difference between nodes and the free surface [39], with the amplitude of surface fluctuations being reflected by the difference between the highest and lowest points on the mold’s free surface. The relative height difference Δ*h* in the model can be determined using Equation (33). As shown in the figure, the overall trend in the free surface wave height distribution is consistent across different SEN depths. The wave height near the SEN and the narrow face exhibits upward fluctuations, while the lowest points of surface fluctuation are located at the 1/4 and 3/4 positions along the wide face of the mold, where the surface waves move downward. It is also evident that the surface fluctuations are the most pronounced near the narrow face, primarily driven by the Z-direction velocity component of the molten steel, indicating that the Z-direction velocity component is the highest in this region. Comparing the free surface fluctuations at different SEN depths, it is observed that as the SEN depth increases from 110 mm to 170 mm, the maximum fluctuation value gradually increases. However, when the SEN depth further increases from 170 mm to 190 mm, the maximum surface fluctuation decreases. This indicates that as the upper recirculation reaches the surface, the Z-direction velocity initially increases with an increasing SEN depth and then decreases, which is consistent with the previously discussed pattern of surface flow velocity.

(33) $$\Delta h=\frac{p_{local}-p_{mean}}{g(\rho_{metal}-\rho_{slag})}$$

where Δ*h* represents the relative height difference in the molten steel at the free surface (m), *p*_local_ denotes the pressure at the nodes on the free surface, and *p*_mean_ is the average pressure across the free surface (Pa). *g* is the gravitational acceleration, taken as 9.8 m/s^2^. *ρ*_metal_ and *ρ*_slag_ represent the densities of molten steel (7020 kg/m^3^) and slag (3000 kg/m^3^), respectively.

**Figure 13:**
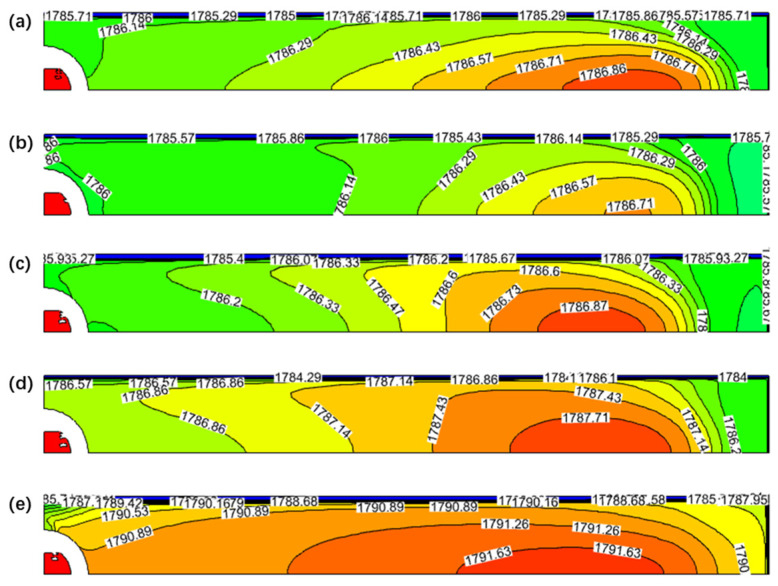
Temperature field distribution of free surface in mold: (**a**) SEN depth of 110 mm; (**b**) SEN depth of 130 mm; (**c**) SEN depth of 150 mm; (**d**) SEN depth of 170 mm; and (**e**) SEN depth of 190 mm.

Figure 13 shows the temperature field distribution of the mold’s free surface at different SEN depths. As seen in the figure, the temperature fields within the mold are quite similar across different SEN depths. However, the temperature distribution on the free surface at an SEN depth of 190 mm differs slightly from the others. Due to the more fully developed upper recirculation, the high-temperature region expands and shifts closer to the 1/4 position of the mold, which is more conducive to the uniform melting of the mold flux.

**Figure 14:**
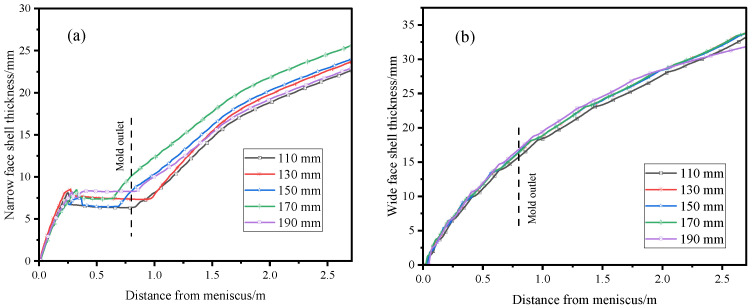
Distribution of shell thickness along casting direction on wide and narrow faces of mold at different SEN depths: (**a**) narrow face; (**b**) wide face.

Figure 14 illustrates the solidification process of the slab on the wide and narrow faces of the mold. The figure shows that the growth of the primary shell on the wide face is less affected by the molten steel flow at different SEN depths, and thus, the shell growth on the wide face largely follows the square root law [40]. At SEN depths of 110 mm, 130 mm, 150 mm, 170 mm, and 190 mm, the thicknesses of the solidified shell at the center of the narrow face at the outlet of the computational domain are 22.6 mm, 23.6 mm, 23.9 mm, 25.6 mm, and 22.9 mm, respectively, exhibiting an initial thickening trend followed by a thinning one. When the slab is at a distance of 0.25–0.75 m from the meniscus, the growth of the solidified shell slows down, entering a “plateau period”. This is due to the direct impact of the side-port jet on the narrow face, which slows down or even remelts the shell at the impact location. It is notable that at an SEN depth of 170 mm, the “plateau period” during the shell growth on the narrow face is the shortest, which is beneficial for the uniform growth of the slab shell, reducing the occurrence of longitudinal cracks and breakout accidents.

## 4. Conclusions

This study focuses on slab molds with dimensions of 1600 mm × 220 mm and 2065 mm × 220 mm, establishing a physical water model with a scaling factor of 0.5 and a coupled flow–heat transfer–solidification numerical model. The impact of the submerged entry nozzle (SEN) depth on the free surface level state, internal flow field, temperature field, and shell solidification was investigated using both the water model and the numerical model. The main conclusions are as follows:

1. Based on the experimental and simulation results, it was found that when the SEN depth is too shallow (80 mm), there are significant fluctuations at the slag–metal interface, and the slag layer on the narrow face is thin, increasing the likelihood of slag entrapment. As the SEN depth increases, the surface flow velocity initially rises and then decreases. There seems to be a critical SEN depth (170 mm in this study) at which the surface flow velocity reaches its maximum, significantly raising the risk of shear-induced slag entrapment. In this study, when the SEN depth is 110 mm, the slag layer on the surface is the most stable and uniform.
2. As the SEN depth increases, the jet impingement point on the narrow face gradually moves downward, and the vortex in the upper recirculation zone evolves from an indistinct circulation to a well-defined vortex with a centered core. When the SEN depth increases to 170 mm, the upper recirculation zone becomes more fully developed, leading to an increase in the maximum surface velocity. However, with further increases in the SEN depth, the kinetic energy loss in the upper recirculation increases, causing the maximum surface velocity to gradually decrease.
3. Within a certain range, increasing the SEN depth can expand the high-temperature region near the meniscus, facilitating the uniform melting of the mold flux. The thickness of the shell on the narrow face is significantly affected by the SEN depth as the high-temperature jet from the side ports impacts the narrow face shell, leading to slow growth or even remelting in the impact zone. Changes in the SEN depth can affect the duration of the “plateau period” in the growth of the narrow face shell, which has a substantial impact on casting quality defects and the risk of breakout.

The optimal SEN depth should be determined as a comprehensive decision-making interval that satisfies all constraints, essentially constituting a linear programming problem. In actual production, various factors such as meniscus flow characteristics, the slag entrapment risk, mold flux melting, impingement depth, internal flow and temperature fields within the mold, and shell uniformity should be considered. This study suggests avoiding the critical SEN depth point; the slag line should either be adjusted to a slightly shallower position (around 110 mm) or to a deeper position (around 190 mm).

## Figures and Tables

**Table 1:** Mold parameters and process parameters.

Parameters	Experimental Simulation	Numerical Simulation
Section size/(mm × mm)	1600 × 220	2065 × 220
Effective height of mold/mm	900	800
Immersion depth of nozzle/mm	80, 110, 140, 170, 200	110, 130, 150, 170, 190
Inner diameter of nozzle/mm	63	63
Outer diameter of nozzle/mm	120	125
Port height and width/mm	70 × 48	70 × 48
Casting speed/(m·min^−1^)	0.9, 1.0, 1.1, 1.2	1.15
Port angle	15 degrees downwards	15 degrees downwards

**Table 2:** Thermal physical properties.

Thermal Physical Properties	Value
Density of mushy zone/(kg·m^−3^)	7200
Viscosity of molten steel/(kg·m^−1^·s^−1^)	0.0055
Liquidus temperature/K	1786
Solidus temperature/K	1752
Specific heat capacity/(J·kg^−1^·K)	720
Latent heat of solidification/(J·kg^−1^)	270,000
Superheat/K	29

## Data Availability

The original contributions presented in this study are included in the article, further inquiries can be directed to the corresponding authors.
